# Growth differentiation factor-15 in patients with or at risk of heart failure but before first hospitalisation

**DOI:** 10.1136/heartjnl-2023-322857

**Published:** 2023-08-11

**Authors:** Joshua Bradley, Erik B Schelbert, Laura J Bonnett, Gavin A Lewis, Jakub Lagan, Christopher Orsborne, Pamela Frances Brown, Nicholas Black, Josephine H Naish, Simon G Williams, Theresa McDonagh, Matthias Schmitt, Christopher A Miller

**Affiliations:** 1 Division of Cardiovascular Sciences, The University of Manchester, Manchester, UK; 2 Manchester University NHS Foundation Trust, Manchester, UK; 3 Department of Medicine, University of Pittsburgh School of Medicine, Pittsburgh, Pennsylvania, USA; 4 Cardiovascular Magnetic Resonance Center, UPMC, Pittsburgh, Pennsylvania, USA; 5 Department of Health Data Science, University of Liverpool, Liverpool, UK; 6 Cardiovascular Division, King's College Hospital, London, UK

**Keywords:** Heart failure, RISK STRATIFICATION

## Abstract

**Objective:**

Identification of patients at risk of adverse outcome from heart failure (HF) at an early stage is a priority. Growth differentiation factor (GDF)-15 has emerged as a potentially useful biomarker. This study sought to identify determinants of circulating GDF-15 and evaluate its prognostic value, in patients at risk of HF or with HF but before first hospitalisation.

**Methods:**

Prospective, longitudinal cohort study of 2166 consecutive patients in stage A–C HF undergoing cardiovascular magnetic resonance and measurement of GDF-15. Multivariable linear regression investigated determinants of GDF-15. Cox proportional hazards modelling, Net Reclassification Improvement and decision curve analysis examined its incremental prognostic value. Primary outcome was a composite of first hospitalisation for HF or all-cause mortality. Median follow-up was 1093 (939–1231) days.

**Results:**

Major determinants of GDF-15 were age, diabetes and N-terminal pro-B-type natriuretic peptide, although despite extensive phenotyping, only around half of the variability of GDF-15 could be explained (R^2^ 0.51). Log-transformed GDF-15 was the strongest predictor of outcome (HR 2.12, 95% CI 1.71 to 2.63) and resulted in a risk prediction model with higher predictive accuracy (continuous Net Reclassification Improvement 0.26; 95% CI 0.13 to 0.39) and with greater clinical net benefit across the entire range of threshold probabilities.

**Conclusion:**

In patients at risk of HF, or with HF but before first hospitalisation, GDF-15 provides unique information and is highly predictive of hospitalisation for HF or all-cause mortality, leading to more accurate risk stratification that can improve clinical decision making.

**Trial registration number:**

NCT02326324.

**WHAT IS ALREADY KNOWN ON THIS TOPIC**

- Circulating growth differentiation factor (GDF)-15 has emerged as a potentially useful prognostic biomarker in heart failure (HF); however, its value has not been assessed in the context of contemporary deep phenotyping.

**WHAT THIS STUDY ADDS**

- Major determinants of GDF-15 level include age, diabetes and NT-pro BNP.
- However, half the variance in circulating levels of GDF-15 remains unexplained despite comprehensive phenotyping.

**HOW MIGHT THIS AFFECT RESEARCH, PRACTICE OR POLICY**

- Including GDF-15 in risk prediction modelling results in more accurate risk stratification that can improve clinical decision making.
- GDF-15 may help to identify patients at risk of adverse outcomes from HF at an early stage, potentially enabling preventative intervention.

## Introduction

One in five middle-aged people will develop heart failure (HF), and, as its risk factors become more prevalent, the burden of HF is expected to increase substantially.^1 2^ Despite improvements in therapies, morbidity and mortality remain high. In particular, hospitalisation for HF (HHF) portends an extremely poor prognosis and accounts for over two-thirds of the economic costs.^2 3^ Identifying people who are at risk of HHF and death is, therefore, a priority, particularly before HHF has occurred.^4–6^

Growth differentiation factor (GDF)-15 was originally characterised as a divergent member of the transforming growth factor-β superfamily, but has more recently been reclassified as a member of the glial cell line-derived neurotrophic factor family, with activity dependent on the tyrosine kinase transmembrane receptor RET (REarranged during Transfection).^7^ In health, GDF-15 expression is predominantly limited to the placenta and prostate, although it is ubiquitously expressed at low levels in most organs and tissues. Stressors such as ischaemia, inflammation, mechanical strain and oxidative stress induce its expression in cells such as cardiomyocytes, endothelial and vascular smooth muscle cells, adipocytes and macrophages.^8^

Circulating GDF-15 has emerged as a potentially useful prognostic biomarker in HF^9–12^; however, its value has not been assessed in the context of contemporary deep phenotyping with cardiovascular magnetic resonance (CMR) imaging, and its evaluation has predominantly been confined to patients with reduced left ventricular (LV) ejection fraction and with advanced disease. Determinants of GDF-15, and thus whether it provides unique information, also remain unclear.

We previously developed and externally validated a risk prediction model that provides accurate, individualised estimates of the risk of HHF and all-cause mortality in patients at risk of HF or with HF but before first hospitalisation.^13^ In the current study, we utilised patients from the same cohort in order to (1) identify determinants of GDF-15 and establish whether GDF-15 provides unique information, and (2) investigate whether GDF-15 provides incremental prognostic value beyond known factors.

## Methods

### Study population

Between 1 June 2016 and 31 May 2018, consecutive patients in American College of Cardiology/American Heart Association (ACC/AHA) stage A to C HF,^6^ undergoing CMR at Manchester University NHS Foundation Trust, UK, were prospectively recruited (NCT02326324). Patients also undergoing measurement of GDF-15 were included in the current analysis. Exclusion criteria included previous hospital admission for HF or a diagnosis of any of the following: amyloidosis, complex congenital heart disease, Fabry disease, hypertrophic cardiomyopathy, iron-overload, myocarditis and stress-induced cardiomyopathy. Patients were also excluded if their CMR scan was not suitable for analysis. The investigation conforms with the principles outlined in the Declaration of Helsinki.

### Procedures

Data were managed using Research Electronic Data Capture (REDCap) hosted at Manchester University NHS Foundation Trust.^14^ Demographics, medical history and ECG indices were determined from medical records. CMR imaging was performed using two scanners (1.5T Avanto, and 3T Skyra; Siemens Medical Imaging). Scanning included steady-state free precession cine imaging in standard long-axis and short-axis planes, basal and mid-LV short-axis T1 mapping (MOdified Look-Locker Inversion Recovery) before and after gadolinium-based contrast agent (gadoterate meglumine (Dotarem; Guerbet, France)), and late gadolinium enhancement (LGE) imaging. CMR image analysis was performed using cvi42 (V.5.6.7; Circle Cardiovascular Imaging; Calgary, Canada) in line with Societal Recommendations as described previously.^13 15 16^ N-terminal pro-B-type natriuretic peptide (NT-proBNP) and GDF-15 (cobas e411 immunoanalyser, Roche Diagnostic, UK) were laboratory assessed from blood sampling performed on the same day as CMR. Further information regarding blood sample processing is provided in the online supplemental material. All baseline data collection, including CMR analysis, were performed prior to receiving, and therefore blinded to, outcome data.

10.1136/heartjnl-2023-322857.supp1 Supplementary data

### Outcome

The primary outcome for the prognostic modelling was a composite of first HHF or all-cause mortality occurring after CMR imaging. Outcome data were obtained from NHS Digital (https://digital.nhs.uk). Hospital Episode Statistics-Admitted Patient Care records were used to identify episodes of hospital admission, and mortality status was derived from Hospital Episode Statistics-Office of National Statistics (Civil registration) data, as described previously.^13^ The follow-up period was from the beginning of recruitment (1 June 2016) until 19 August 2020. Time to the composite endpoint was defined as the time to the first event or censored at the date of end of follow-up if no event occurred. NHS Digital provided outcome data blinded to participant characteristics.

### Statistical analysis

All analyses were performed using R (V.4.2.0, R Foundation for Statistical Computing, Austria). Missing data were handled using multiple imputation with chained equations.^13,17^ Model features were combined and pooled according to current guidelines.^18 19^ Candidate predictors were identical to those in the final previously published model, that is, age, female sex, white race, body mass index, percutaneous coronary intervention, coronary artery bypass grafting, stroke, diabetes, hypertension, raised cholesterol, chronic obstructive pulmonary disease, atrial fibrillation, current or past smoking, estimated glomerular filtration rate, NT-proBNP, indexed LV mass, global longitudinal strain, myocardial infarction, non-ischaemic LGE and myocardial extracellular volume (ECV).^13^ Univariable and multivariable linear regression were conducted to investigate the relationships between baseline variables and natural logarithmic transformed (ln) GDF-15. ln GDF-15 was used because GDF-15 data had a non-normal distribution (online supplemental figure 1). A multivariable linear regression model was developed using backward stepwise Akaike information criterion (AIC) selection.^17^

The prognostic value of GDF-15 was determined using Cox proportional hazards modelling on a time-since-study-entry timescale, as previously described.^13^ Prognostic modelling additionally considered (ln) GDF-15 as a candidate predictor. Further details are provided in the online supplemental material. The incremental prognostic value of GDF-15 was evaluated descriptively, via continuous Net Reclassification Improvement, and using decision curve analysis.^20–23^ To allow appropriate comparison with the previously validated risk prediction model, the original model was rederived in the current study cohort.

### Patient and public involvement

The study was part of a research programme with a dedicated patient advisory group, that was identified and first met during set up. The group comprised six patients and was chaired by a patient. The advisory group was involved with study design and management, meeting annually throughout the study to provide input on study progress and management. The study team also discussed preliminary results with the group, to get their input on the interpretation of the findings.

## Results

### Patients and outcome

**Figure 1:**
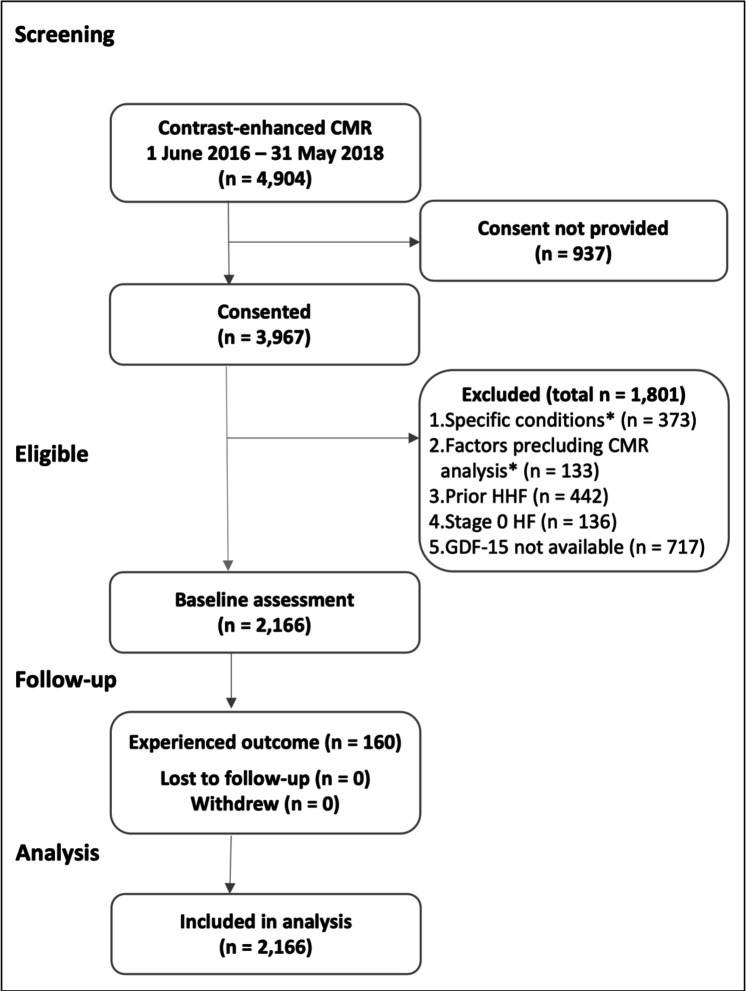
STROBE diagram. *Specific conditions: acute myocarditis (n=15), amyloidosis (n=20), complex congenital heart disease (n=12), Fabry disease (n=39), hypertrophic cardiomyopathy (n=266), iron overload (n=7), stress-induced cardiomyopathy (n=14). Factors precluding CMR analysis: abandoned scanning (n=59), artefact (n=14), incomplete scan availability (n=60). CMR, cardiovascular magnetic resonance; GDF-15, growth differentiation factor-15; HF, heart failure; HHF, hospitalisation for heart failure.

The cohort comprised 2166 participants (figure 1). Baseline characteristics are summarised in table 1. Median follow-up duration was 1093 (939–1231) days. The composite outcome of HHF or all-cause mortality occurred in 160 participants (7.4%, annualised rate 2.5%). Seventy-one patients experienced HHF, and 89 died. No patients were lost to follow-up.

**Table 1:** Baseline characteristics

Baseline characteristic	n=2883	Missing (%)
Age	56.5±15.5	0
Male	1388 (64%)	0
Ethnicity		0
White	1808 (83.5%)	
Asian	105 (4.8%)	
Black	55 (2.5%)	
Other	35 (1.6%)	
Not declared/prefer not to say	163 (7.5%)	
Body mass index (kg/m^2^)	28.8±6.0	27 (1.2)
Heart failure stage		0
A	615 (28.4%)	
B	551 (25.4%)	
C	1000 (46.2%)	
D	0	
Referring centre		10 (<1)
Cardiac centre	934 (43.3%)	
District hospitals	1222 (56.7%)	
Previous revascularisation	448 (20.7%)	0
PCI	308 (14.2%)	0
CABG	140 (6.5%)	0
Stroke or TIA	148 (6.8%)	0
Peripheral vascular disease	83 (3.8%)	0
Diabetes	313 (14.4%)	0
Hypertension	1021 (47.1%)	0
Raised cholesterol	991 (45.8%)	0
COPD	128 (5.9%)	0
Atrial fibrillation	328 (15.1%)	0
History of smoking	1107 (51.1%)	0
Heart rate (bpm)	66.8±12.7	8 (<1)
QRS duration (ms)	103±21.9	283 (13)
eGFR (mL/min)	78±13.0	18 (<1)
NT-proBNP (pg/mL)	137.2 (56.5–444.4)	7 (<1)
GDF-15 (pg/mL)	1099.5 (717.2–1755)	0
LVEF (%)	56.1±12.1	3 (<1)
Indexed LV EDV (mL/m^2^)	89.6±27.0	7 (<1)
Indexed LV ESV (mL/m^2^)	41.3±24.3	7 (<1)
Indexed LV mass (g/m^2^)	58.9±17.9	7 (<1)
LV global longitudinal strain (%)	−17.53±4.42	65 (3)
Infarct LGE	494 (22.8%)	0
Atypical (non-infarct) LGE	349 (16.1%)	0
Myocardial ECV (%)	26.1±3.4	165 (7.6)
Composite outcome	160 (7.4%)	0
First hospitalisation for heart failure	71 (3.3%)	0
All-cause mortality	89 (4.1%)	0
Time to first hospitalisation for heart failure, all-cause mortality or censor	1093 (939–1231)	0

Data are n (%), n/N (%), mean±SD or median (IQR).

CABG, coronary artery bypass grafting; COPD, chronic obstructive pulmonary disease; ECG, electrocardiogram; ECV, extracellular volume; EDV, end-diastolic volume; eGFR, estimated glomerular filtration rate; ESV, end-systolic volume; GDF-15, growth differentiation factor-15; Indexed, indexed to body surface area; LGE, late gadolinium enhancement; NT-proBNP, N-terminal pro-B-type natriuretic peptide; PCI, percutaneous coronary intervention; TIA, transient ischaemic attack.

### Determinants of GDF-15

Median GDF-15 was 1099.5 pg/mL (717.2− 1755.0). Univariable associations between baseline variables and GDF-15, and the adjusted R^2^ for each variable, are presented in online supplemental table 1. Independent determinants of GDF-15 are presented in table 2. The strongest predictors of GDF-15 were age, diabetes and NT-proBNP. The adjusted R^2^ for the multivariable model was 0.51.

**Table 2:** Multivariable determinants of natural logarithmic transformed GDF-15

Term	β-Coefficient (SE)	95% CI	t-Statistic	P value
(Intercept)	5.677 (0.140)	5.403 to 5.951	40.630	<0.0001
Age (years)	0.015 (0.001)	0.013 to 0.016	17.189	<0.0001
Female sex	−0.139 (0.024)	−0.186 to −0.091	−5.702	<0.0001
Body mass index (kg/m^2^)	0.004 (0.002)	0.001 to 0.008	2.487	0.013
Stroke	0.084 (0.041)	0.004 to 0.163	2.061	0.039
Diabetes	0.420 (0.030)	0.360 to 0.480	13.788	<0.0001
Hypertension	0.092 (0.024)	0.046 to 0.139	3.898	<0.0001
Raised cholesterol	−0.055 (0.024)	−0.103 to −0.008	−2.273	0.023
COPD	0.200 (0.044)	0.113 to 0.286	4.535	<0.0001
Atrial fibrillation	−0.061 (0.029)	−0.119 to −0.004	−2.097	0.036
Ever smoker	0.062 (0.021)	0.021 to 0.103	2.992	0.003
eGFR (mL/min)	−0.005 (0.001)	−0.007 to −0.004	−6.237	<0.0001
ln (NT-proBNP) (log pg/mL)	0.133 (0.009)	0.115 to 0.150	14.759	<0.0001
Indexed LV mass (g/ m^2^)	−0.003 (0.001)	−0.004 to −0.001	−4.039	<0.0001
Infarct LGE	0.041 (0.027)	−0.011 to 0.094	1.553	0.12
Myocardial ECV (%)	0.009 (0.004)	0.002 to 0.016	2.663	0.008

COPD, chronic obstructive pulmonary disease; ECV, extracellular volume; eGFR, estimated glomerular filtration rate; GDF-15, growth differentiation factor-15; Indexed, indexed to body surface area; LGE, late gadolinium enhancement; NT-proBNP, N-terminal pro-B-type natriuretic peptide; TIA, transient ischaemic attack.

### Relationship between GDF-15 and outcome

In multivariable prognostic modelling, GDF-15 was the strongest predictor of the composite primary outcome of HHF or all-cause mortality (HR 2.12, 95% CI 1.71 to 2.63; p<0.0001; table 3). GDF-15 was also strongly and independently predictive of the individual components of the primary outcome: HHF (HR 1.92, 95% CI 1.33 to 2.79; p=0.0008; online supplemental table 2) and all-cause mortality (HR 2.74, 95% CI 2.06 to 3.63; p<0.0001; online supplemental table 2).

### Incremental prognostic value of GDF-15

Including GDF-15 as a candidate variable resulted in a more parsimonious multivariable prognostic model than when GDF-15 was not considered (six variables rather than seven) (table 3). Performance of the model that includes GDF-15 was very good, with marginally higher discrimination (c-index 0.82; 95% CI 0.80 to 0.85) than when GDF-15 was not considered (0.80; 95% CI 0.77 to 0.83). Estimates of calibration from resampling indicate a high degree of agreement between predicted and observed risk for >90% of cases (online supplemental figure 2).

**Table 3:** Multivariable prognostic modelling for the composite outcome

	Model that did not consider GDF-15 as a candidate variable				Model that did consider GDF-15 as a candidate variable			
Term	HR	95% CI	Wald χ^2^	P value	HR	95% CI	Wald χ^2^	P value
Age	1.026	1.014 to 1.037	19.344	<0.0001	1.015	1.002 to 1.029	4.953	0.024
Diabetes	1.437	1.078 to 1.917	6.171	0.014	–	–	–	–
COPD	1.742	1.239 to 2.449	10.296	0.0015	1.556	1.051 to 2.304	5.208	0.028
ln (NT-proBNP)	1.275	1.118 to 1.455	13.455	0.0004	–	–	–	–
ln (GDF-15)	–	–	–	–	2.122	1.710 to 2.634	47.377	<0.0001
GLS	1.073	1.038 to 1.109	17.526	<0.0001	1.099	1.063 to 1.136	31.339	<0.0001
Infarct LGE	1.560	1.196 to 2.036	10.851	0.0012	1.452	1.064 to 1.981	5.625	0.019
Myocardial ECV	1.083	1.044 to 1.122	18.844	<0.0001	1.082	1.039 to 1.126	15.146	0.0002

The multivariable model that did not consider GDF-15 as a candidate variable comprised the following independent variables: age, diabetes, COPD, ln (NT-proBNP), GLS, infarct LGE and myocardial ECV. The multivariable model that did consider GDF-15 as a candidate variable comprised following independent variables: age, COPD, ln (GDF-15), GLS, infarct LGE and myocardial ECV.

COPD, chronic obstructive pulmonary disease; ECV, extracellular volume; GDF-15, growth differentiation factor-15; GLS, global longitudinal strain; LGE, late gadolinium enhancement; NT-proBNP, N-terminal pro-B-type natriuretic peptide.

**Figure 2:**
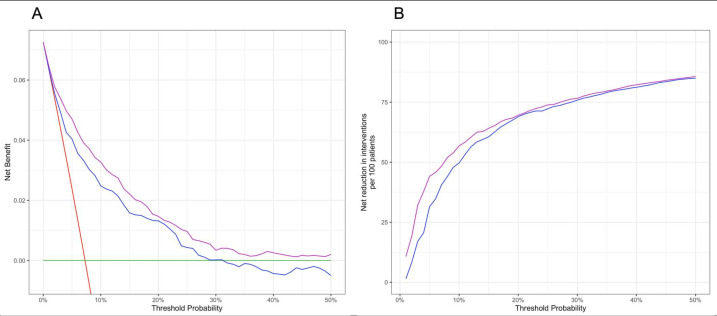
Incremental prognostic value of GDF-15. (A) Decision curve analysis shows that risk prediction using the model that included GDF-15 (purple line) leads to net clinical benefit at all decision thresholds (ie, the threshold at which a decision regarding patient management, such as commencing an intervention to reduce risk, is taken) compared with the model that did not consider GDF-15 (blue). By convention, the default strategies of intervening for all patients (red) and intervening for none (green), are also shown. (B) Furthermore, risk prediction using the model that included GDF-15 (purple line) leads to a net reduction in the number of unnecessary interventions compared with the model that did not consider GDF-15 (blue). GDF-15, growth differentiation factor-15.

Net Reclassification Improvement analysis demonstrated that the predictive accuracy of the model that includes GDF-15 (comprised of the variables: age, COPD, ln (GDF-15), GLS, infarct LGE and myocardial ECV) was significantly higher than the model not considering GDF-15 (comprised of the variables: age, diabetes, COPD, ln (NT-proBNP), GLS, infarct LGE and myocardial ECV) (continuous Net Reclassification Improvement 0.26; 95% CI 0.13 to 0.39; online supplemental table 3). Similarly, decision curve analysis showed that risk prediction using the model that includes GDF-15 was superior across the full spectrum of risk (figure 2). As an illustration, using an example decision threshold (ie, the threshold at which a decision regarding patient management, such as commencing an intervention to reduce risk, is taken) for 3-year risk of HHF or all-cause mortality of 10%, in a population with 73 events per 1000 person years (ie, a population with a similar prevalence to the development cohort), the prognostic model that includes GDF-15 would identify 8 (11%) additional true events, without increasing the number of false-positive predictions, while also reducing the number of patients receiving unnecessary interventions by 5 (7%), without increasing the number of false-negative predictions. Thus overall, the model that includes GDF-15 would lead to benefit in an additional 18% of patients compared with the model not considering GDF-15 (ie, featuring NT-proBNP).

## Discussion

The principal findings of this study are that GDF-15 was the strongest predictor of HHF or all-cause mortality in patients at risk of HF or with HF but before first hospitalisation. Moreover, GDF-15 resulted in a more parsimonious prognostic model, with higher predictive accuracy and which would lead to greater clinical benefit across the entire risk spectrum. Several determinants of GDF-15 were identified, however, despite comprehensive phenotyping, only around half of the variability in GDF-15 could be explained, indicating that GDF-15 provides unique information.

Studies evaluating the prognostic value of biomarkers in HF typically include a high proportion of patients with quite advanced disease and who have previously been hospitalised for HF.^24 25^ While such studies are important, greater potential patient, societal and economic benefit could be derived from identifying patients at risk of adverse outcome from HF at an early stage; when the underlying disease mechanisms are more likely modifiable, relative health can be maintained and the financial costs associated with HHF have not already been incurred. Indeed, as the population ages and cardiometabolic conditions that predispose HF become more prevalent, early risk stratification to facilitate preventative strategies is likely to become an even greater priority.

It is with these factors in mind that the current study focused on patients at a relatively early stage in the HF process. More than 50% of the cohort were at risk of HF or had pre-HF (ACC/AHA stage A or B), and patients with ACC/AHA stage C HF who had experienced previous HHF were excluded. As a result, median NT-proBNP (137 pg/mL (56.5–444.4)) and annualised outcome rate (2.5% /year) were relatively low.

In this context, GDF-15 was independently predictive of HHF or all-cause mortality; indeed, the HR and Wald χ^2^ indicate that it was the strongest predictor. GDF-15 was also a strong independent predictor of both HHF and all-cause mortality individually.

A recent meta-analysis summarises the prognostic importance of GDF-15 in HF.^26^ In a total of 10 eligible studies, including 6244 patients with established HF, a 1 lnU increase in baseline GDF-15 was associated with a 6% increase in all-cause mortality after multivariable adjustment (HR 1.06, 95% CI 1.03 to 1.10, p<0.001).^26^ The prognostic significance was greater in patients with reduced (<50%) versus preserved (≥50%) LVEF (HR 1.46 and 1.008, respectively), and ischaemic versus non-ischaemic aetiology (HR 1.75 and 1.01, respectively).^26^ There are several important differences between our study and the studies included in the meta-analysis; the latter are limited by small sample size and included patients with established symptomatic HF and predominantly patients with reduced LVEF. Our study focused on patients at an early stage of the disease, either patients at-risk of HF or patients with HF before first hospitalisation. It is noteworthy that GDF-15 was a more significant predictor of all-cause mortality in our early at-risk HF cohort (HR 2.74) compared with the established HF cohort in the meta-analysis (HR 1.06).^26^ In the current study, participants also underwent deep phenotyping with baseline CMR evaluation. We were therefore able to include and adjust for myocardial fibrosis, global longitudinal strain and LGE. Finally, our study is the first to use decision curve analysis to assess the incremental value of GDF-15 to guide treatment decisions in early HF. If interventions are developed which prove efficacious in treating or preventing early HF (such as lifestyle modification or medical therapy), then a predictive model will be necessary to identify which patients would benefit from these interventions in this relatively low risk cohort. Figure 2 demonstrates that the addition of GDF-15 to the prognostic model would result in more patients being appropriately treated, and fewer patients inappropriately treated, across the full spectrum of treatment thresholds.

Interestingly, the addition of GDF-15 to the prognostic model resulted in NT-proBNP and diabetes becoming non-significant as predictors of first HHF and all-cause mortality. GDF-15 shares significant univariable and multivariable associations with both NT-proBNP and diabetes, so this may be explained by collinearity between the variables.

An important observation from this study is that GDF-15 was a stronger predictor of all-cause mortality than HHF (HR 2.74 and 1.92, respectively). Chan *et al* 10 demonstrated the same result with competing risk analysis. This supports the view that GDF-15 is a prognostic marker of adverse cardiometabolic risk more generally rather than specifically for HF per se. For instance, in patients with type 2 diabetes, GDF-15 was predictive of adverse cardiovascular (myocardial infarction, stroke and cardiovascular death) and renal outcomes (40% decline in estimated glomerular filtration rate, end-stage renal failure and renal death), in addition to HF.^27^ In outpatients with cardiovascular risk factors, GDF-15 was predictive of all-cause mortality and stroke.^28^ This hypothesis is also biologically plausible. GDF-15 is released by a wide variety of cell types (skeletal and cardiac muscle, vascular endothelial cells, fibroblasts and immune cells) in response to tissue injury, physiological stress and ageing.^29^ GDF-15 has been shown to have a key role in regulating mitochondrial function, cellular metabolism and oxidative stress.^29^ It is therefore likely that GDF-15 is involved in many cardiovascular and non-cardiovascular disease states.

In keeping with the nature of the cohort studied, circulating levels of GDF-15 in the current study (1099.5 pg/mL (717.2–1755.0)) were higher than that reported in healthy populations (762 ng/L (600–959)),^30^ but lower than that in more established HF (eg, 1626 ng/L (1159–2398) at baseline in the sacubitril/valsartan group in the PARADIGM‐HF trial^12^). Major determinants of GDF-15 were age, diabetes, NT-proBNP, renal function and hypertension, which are in keeping with the findings of previous studies.^9–12^ Additionally, myocardial fibrosis, measured using CMR ECV, was an independent determinant of GDF-15, which is in keeping with the study by Lok *et al*,^31^ who, in a small group of patients with advanced non-ischaemic cardiomyopathy, found GDF-15 to be moderately correlated with histological collagen volume (r=0.61, p=0.01).

Reflecting the comprehensive phenotyping in the current study, the adjusted R^2^ for the multivariable model (0.51) was substantially higher than in previous studies investigating determinants of GDF-15 (eg, 0.39 in the study by Bouabdallaoui *et al* 12). Nevertheless, even despite the comprehensive phenotyping, only around half of the variability of GDF-15 could be explained, demonstrating that, rather than representing an integrated biomarker of multiple co-morbidities as has been postulated, GDF-15 provides unique information. Further work is required to understand its expression.

A limitation of the study is the lack of external validation of the prognostic model that includes GDF-15. This represents an aim for future work. Participants were undergoing CMR, thus the population is potentially skewed; however, the CMR service at Manchester University NHS Foundation Trust serves a wide range of hospitals across the North West of England (56.7% of patients were from district hospitals), thus, in this regard, the current study is more representative than many previous studies evaluating GDF-15 in HF. Furthermore, the substantially higher precision that CMR provides for standard cardiac measurements, as well as the more contemporary assessments of cardiac injury and adaptation that CMR provides, allows for more detailed and accurate interrogation of the prognostic role of GDF-15 and its determinants.

In conclusion, in patients at risk of HF, or with HF but before first hospitalisation, GDF-15 provides unique information and is highly predictive of HHF or all-cause mortality, leading to more accurate risk stratification that can improve clinical decision making.

## Data Availability

Data are available upon reasonable request. Deidentified participant data will be made available to requestors one year after date of publication, with no end date to availability. Data will be shared after an appropriate proposal is submitted and may be used for any purpose. Proposals should be directed to christopher.miller@manchester.ac.uk. Requestors will be required to sign a data access agreement.
