# Supplementary material for: Growth differentiation factor-15 in patients with or at risk of heart failure but before first hospitalisation
Source: Heart. 2023 Aug 11;110(3):195–201. doi: 10.1136/heartjnl-2023-322857 (PMC10850645; doi:10.1136/heartjnl-2023-322857)
Supplement: Supplementary data [file heartjnl-2023-322857supp001.pdf]

GDF-15 in patients with or at risk of heart failure but before first hospitalisation

Supplemental Material

Supplemental Methods

Blood sample processing

N-terminal pro-B-type natriuretic peptide (NT-proBNP and growth differentiation factor-15 (GDF-15) were laboratory assessed on **cobas e** 411 immunoanalyser (Roche Diagnostics, UK). A representative description of the full procedure is as follows: in patients who specifically consented for blood sample analysis and storage, 10mL EDTA blood sample was drawn on the same day as CMR from the same canula inserted to administer the gadolinium-based contrast agent required for scanning. The collected samples were stored at 2 - 8°C until same day sampling. Samples were centrifuged at 3000RPM for 10 minutes at 4°C. Four plasma aliquots of 500µL were drawn and subsequently stored, along with the remaining blood pellet, at -80°C. On the day of assay, one aliquot per patient was thawed at 20°C for one hour prior to assay. Measurement of all assays was undertaken concurrently during a single first thaw of stored serum.

Each assay was calibrated and quality controlled using the manufacturer's reagents. Calibration was performed for each new reagent lot and as indicated by quality control findings, or every seven days per reagent kit (whichever occurred first), thereafter. Quality control was performed on the day of, and prior to, assay. Assay characteristics were determined by the manufacturer (see Supplemental Table 4). Assay variability of NT-proBNP (Supplemental Table 5a and 5b) and GDF-15 (Supplemental Table 6) were determined by the manufacturer and are reported as precision. Results outside the measuring range were reported as the measuring range limit for continuous analysis.

Statistical analyses

Multiple imputation

Imputation from the previous investigation was preserved for consistency with the original findings (13). Missing data were unintentional and their absence was due to incomplete medical records, incomplete CMR, or blood sampling not being done; thus, data were assumed to be missing at random. Multiple imputation by chained equations was used to create 20 imputed datasets (17,19). In the previous investigation, baseline characteristics were available for 30 covariables. Missing data were imputed from these variables (age, sex, race, Index of Multiple Deprivation, body-mass index, percutaneous coronary intervention, coronary artery bypass graft, stroke or transient ischaemic attack, peripheral vascular disease, diabetes, hypertension, raised cholesterol, chronic obstructive pulmonary disease (COPD), atrial fibrillation, past or current smoker, QRS complex duration, estimated glomerular filtration rate, NT-pro-BNP, *ln* (NT-proBNP), high-sensitivity cardiac troponin T, *ln* (high-sensitivity cardiac troponin T), MRI field strength, left ventricular ejection fraction, indexed myocardial mass, global longitudinal strain, right ventricular ejection fraction, body surface area-indexed left atrial area, myocardial infarction, atypical (non-infarct) late gadolinium enhancement, and myocardial extracellular volume) and 2 outcome variables (the composite outcome and time to composite outcome) by use of predictive mean matching. For the present investigation, the subset of imputation results for those 2,166 patients with complete GDF-15 were selected, and their GDF-15 results were appended to the data.

Cox regression

Variable selection

Candidate predictors were those available in the external validation cohort of the previously published model, i.e.: age, female sex, white race, body mass index, percutaneous coronary intervention, coronary artery bypass grafting, stroke, diabetes, hypertension, raised cholesterol, chronic obstructive pulmonary disease, atrial fibrillation, current or past

smoking, estimated glomerular filtration rate, NT-proBNP, indexed left ventricular mass, global longitudinal strain, myocardial infarction, non-ischaemic LGE and myocardial extracellular volume (ECV) (13), and additionally featured log-transformed GDF-15.

Variables showing a significant ($p<0.05$) univariable relationship with the composite outcome were considered for entry into the multivariable model (Supplemental Table 7). In the presence of multiply imputed data, a composite stepwise selection procedure was utilised to identify the parsimonious multivariable model (13, 17). First, backward stepwise AIC model selection was done separately in each imputed dataset. Variables present in more than 50% of models were then considered for inclusion in the parsimonious model with the multivariable D_1 Wald test ($p<0.05$) (13, 17).

Internal validation

The developed model was validated internally. Non-parametric bootstrapping was used to estimate optimism, and examine model stability. In each of the imputed datasets, the entire modelling process, including predictor variable selection, was repeated in 1,000 bootstrap samples. Using the estimated optimism as a uniform shrinkage factor, all the predictor effects in the final model were penalised in order to account for over-fitting (20). Model performance was evaluated in terms of discrimination and calibration. Discrimination was measured using Harrell's C-index and calibration was evaluated graphically (20). Model features and performance measures were combined across imputed datasets according to current guidelines (19).

Proportional hazards assumption

Schöenfeld residual tests were used to evaluate the proportional hazards assumption (20). Where potential violations were evidenced ($p>0.05$), Schöenfeld residuals for those covariables were investigated graphically. All models satisfied the proportional hazards assumption.

Secondary analyses

Nested modelling

The final model that could have included GDF-15 had a different set of known risk factors in it from the model that wasn't able to select GDF-15. To further investigate the incremental prognostic value of GDF-15, an additional model was developed that included all of the same known risk factors from the original model and GDF-15. Model features, net reclassification improvement indices and decision curve analysis were additionally estimated for these two nested models.

Comparison of Heart Failure Stages A and B versus C

The progression from HF Stages A and B to HF Stage C denotes a key milestone in the progression of patients, whereby Stage C is typically demarcated by the onset of symptoms and/or signs of HF. In order to investigate whether GDF-15 performs differently as a prognostic marker in pre- (i.e. Stage A/B) and overt- (Stage C) HF patients, the prognostic value of GDF-15 was considered both uni- and multivariably for subsets of patients in Stages A/B (n=1,166) and Stage C (n=1,000). The discrimination, as a measure of model performance, was also calculated in each subset (20).

Bradley J, *et al. Heart* 2024; 110:195–201. doi: 10.1136/heartjnl-2023-322857

Supplemental Results

Secondary analyses

Nested modelling

Model features of the nested models are presented in Supplemental Table 8. Net reclassification improvement indices are presented in Supplemental Table 9. Decision curve analysis for the nested models in presented in Supplemental Figure 3. Similar to the results in the main text, these investigations continue to indicate that risk prediction is improved by the inclusion of GDF-15, and that GDF-15 offers incremental prognostic value beyond known risk factors.

Comparison of Heart Failure Stages A and B versus C

The univariable association of natural log-transformed GDF-15 and the composite primary outcome for each of the HF sub-groups are presented in Supplemental Table 10.

In multivariable prognostic modelling, natural log-transformed GDF-15 remained strongly predictive in both A/B and C (see Supplemental Table 11).

The discrimination for the GDF-15 model in each of the subgroups is presented in Supplemental Table 12. In each sub-group, model discrimination was excellent.

Bradley J, *et al. Heart* 2024; 110:195–201. doi: 10.1136/heartjnl-2023-322857

Supplemental tables and figures

Bradley J, *et al. Heart* 2024; 110:195–201. doi: 10.1136/heartjnl-2023-322857

Supplemental Table 1. Univariable determinants of natural logarithmic transformed GDF-15

Term	Intercept	β-Coefficient (standard error)	95 % confidence interval	t statistic	p value	Adjusted R^2
Age	5.649	0.025 (0.001)	0.023 - 0.026	33.340	<0.0001	0.339
Female sex	7.105	-0.129 (0.03)	-0.187 - -0.071	-4.358	<0.0001	0.008
White Race	7.001	0.069 (0.038)	-0.006 - 0.145	1.808	0.071	0.001
Body mass index	6.853	0.007 (0.002)	0.002 - 0.012	3.007	0.0027	0.004
Percutaneous coronary intervention	7.022	0.255 (0.041)	0.176 - 0.335	6.301	<0.0001	0.018
Coronary artery bypass grafting	7.029	0.461 (0.057)	0.348 - 0.573	8.042	<0.0001	0.029
Stroke	7.036	0.336 (0.056)	0.226 - 0.446	5.984	<0.0001	0.016
Diabetes	6.965	0.645 (0.038)	0.570 - 0.720	16.875	<0.0001	0.116
Hypertension	6.859	0.423 (0.027)	0.369 - 0.476	15.564	<0.0001	0.100
Raised cholesterol	6.897	0.352 (0.028)	0.298 - 0.406	12.731	<0.0001	0.069
Chronic obstructive pulmonary disease	7.032	0.456 (0.06)	0.338 - 0.573	7.620	<0.0001	0.026
Atrial fibrillation	7.020	0.258 (0.039)	0.180 - 0.335	6.531	<0.0001	0.019
Ever Smoker	6.995	0.125 (0.028)	0.069 - 0.181	4.400	<0.0001	0.008
Estimate glomerular filtration rate	8.370	-0.017 (0.001)	-0.019 - -0.015	-15.959	<0.0001	0.107
ln (NTproBNP)	5.861	0.236 (0.008)	0.220 - 0.252	28.737	<0.0001	0.277
Indexed myocardial mass	6.838	0.004 (0.001)	0.002 - 0.005	4.696	<0.0001	0.010
Global longitudinal strain	7.764	0.040 (0.003)	0.034 - 0.046	12.815	<0.0001	0.071
Infarct LGE	6.963	0.418 (0.033)	0.354 - 0.482	12.724	<0.0001	0.069
Atypical LGE	7.047	0.074 (0.039)	-0.002 - 0.150	1.913	0.056	0.001
Myocardial ECV	6.200	0.033 (0.004)	0.024 - 0.041	7.629	<0.0001	0.027

ECV=extracellular volume. LGE=late gadolinium enhancement. *ln*=natural logarithm. NT-proBNP=N-terminal pro-B-type natriuretic peptide.

Supplemental Table 2. Multivariable prognostic models, including GDF-15 as a candidate variable, for the individual components of the composite primary outcome

	Hospitalisation for heart failure				All-cause mortality			
**Term**	**Hazard Ratio**	**95% confidence interval**	**Wald χ^2**	**p value**	**Hazard Ratio**	**95% confidence interval**	**Wald χ^2**	**p value**
Age	1.008	0.987 - 1.029	0.528	0.47	1.025	1.006 - 1.043	6.907	0.010
COPD	1.331	0.681 - 2.603	0.727	0.40	1.611	0.94 - 2.762	3.084	0.082
ln (GDF-15)	1.922	1.326 - 2.787	12.362	0.0008	2.736	2.061 - 3.632	49.767	<0.0001
GLS	1.194	1.130 - 1.262	41.087	<0.0001	1.047	1.001 - 1.095	4.087	0.046
Infarct LGE	1.535	0.929 - 2.538	2.906	0.093	1.536	1.003 - 2.352	3.99	0.049
Myocardial ECV	1.092	1.024 - 1.165	7.497	0.0082	1.083	1.025 - 1.144	8.319	0.0049

Abbreviations as per previous table.

Bradley J, *et al. Heart* 2024; 110:195–201. doi: 10.1136/heartjnl-2023-322857

Supplemental Table 3. Net Reclassification Improvement

	Estimate	95% confidence interval
Pr (Up | Case)	0.207	0.145 – 0.274
Pr (Down | Case)	0.793	0.726 – 0.855
Pr (Down | Ctrl)	0.923	0.911 – 0.934
Pr (Up | Ctrl)	0.077	0.066 – 0.089

Pr=probability. Up=individual receives a higher risk estimate in the new model. Down= individual receives a lower risk estimate in the new model. Case=individual experiencing outcome. Ctrl=individual not experiencing outcome.

Bradley J, *et al. Heart* 2024; 110:195–201. doi: 10.1136/heartjnl-2023-322857

Supplemental Table 4. Biomarker assay characteristics

	**NT-proBNP**	**hs-cTnT**	**GDF-15**
Reagent	Elecsys proBNP II	Elecsys Troponin T hs	Elecsys GDF-15
Limit of Blank	3 pg/mL	3 pg/mL	350 pg/mL
Limit of Detection	5 pg/mL	5 pg/mL	400 pg/mL
Limit of Quantitation	50 pg/mL	13 pg/mL	400 pg/mL
Coefficient of variation	20%	10%	20%
Measuring range	5 – 35,000 pg/mL	3 - 10,000 pg/mL	400 – 20,000 pg/mL

The Limit of Blank is the 95th percentile from n ≥ 60 measurements of analyte-free sample over several independent series. The Limit of Blank corresponds to the concentration below which analyte-free samples are found with a probability of 95%.
The Limit of Detection is determined based on the Limit of Blank and the standard deviation of low concentration samples. The Limit of Detection corresponds to the lowest analyte-concentration which can be detected (value above the Limit of Blank with a probability of 95%).
The Limit of Quantitation (function sensitivity) is the lowest analyte concentration that can be reproducibly measured with an intermediate precision equal to the stated coefficient of variation.

Bradley J, *et al. Heart* 2024; 110:195–201. doi: 10.1136/heartjnl-2023-322857

Supplemental Table 5. NT-proBNP assay precision

a)

**cobas e** 411 analyzer					
	**Repeatability**				
Sample	Mean		SD		CV
	pg/mL	pmol/L	pg/mL	pmol/L	%
Human serum 1	44.0	5.19	1.84	0.22	4.2
Human serum 2	126	14.9	3.06	0.36	2.4
Human serum 3	2410	28	31.7	3.74	1.3
Human serum 4	33606	3966	922	109	2.7
PC CARDII1	82.0	9.86	2.11	0.25	2.58
PC CARDII2	2318	274	27.3	3.22	1.18

CV=coefficient of variation. PC CARDII = PreciControl Cardiac II. SD=standard deviation.

b)

**cobas e** 411 analyzer					
	**Intermediate precision**				
Sample	Mean		SD		CV
	pg/mL	pmol/L	pg/mL	pmol/L	%
Human serum 1	44.0	5.19	2.02	0.24	4.6
Human serum 2	126	14.9	3.23	0.38	2.6
Human serum 3	2410	28	44.2	5.22	1.8
Human serum 4	33606	3966	1288	152	3.8
PC CARDII1	82.0	9.86	2.27	0.27	2.8
PC CARDII2	2318	274	36.6	4.32	1.6

Abbreviations per previous tables.

Precision was determined using Elecsys reagents, pooled human sera and controls in a modified protocol (EP5-A) of the CLSI (Clinical and Laboratory Standards Institute): 6 times daily for 10 days (n=60); repeatability on MODULAR ANALYTICS E170 analyer, n=21.

Bradley J, *et al. Heart* 2024; 110:195–201. doi: 10.1136/heartjnl-2023-322857

Supplemental Table 6. GDF-15 assay precision

**cobas e** 411 analyzer					
		**Repeatability**		**Intermediate precision**	
Sample	Mean pg/mL	SD pg/mL	CV %	SD pg/mL	CV %
Human serum 1	460	6.75	1.5	14.0	3.1
Human serum 2	1148	8.61	0.7	29.0	2.5
Human serum 3	1673	20.6	1.2	45.6	2.7
Human serum 4	4952	64.4	1.3	129	2.6
Human serum 5	9720	77.5	0.8	234	2.4
Human serum 6	18690	239	1.3	538	2.9
PC CARDII1	1329	13.9	1.0	31.7	2.4
PC CARDII2	7211	71.5	1.0	176	2.4

Abbreviations per previous tables.

Precision was determined using Elecsys reagents, samples and controls in a protocol (EP05-A3) of the CLSI: 2 runs per day in duplicate each for 21 days (n=84).

Supplemental Table 7. Univariable association with composite outcome.

Term	Hazard Ratio	95% confidence interval	Wald χ^2	P value
Age	1.049	1.036 - 1.063	54.789	0.000
Female sex	0.706	0.500 - 0.997	3.961	0.048
White race	1.155	0.74 - 1.803	0.408	0.524
Body mass index	1.013	0.988 - 1.038	1.024	0.313
PCI	1.091	0.704 - 1.689	0.153	0.696
CABG	1.420	0.817 - 2.468	1.572	0.212
Stroke or TIA	1.727	1.054 - 2.831	4.769	0.030
Diabetes	2.135	1.493 - 3.054	17.544	0.000
Hypertension	1.250	0.914 - 1.708	1.980	0.161
Raised cholesterol	1.247	0.913 - 1.705	1.952	0.164
COPD	3.668	2.432 - 5.534	39.004	0.000
Atrial fibrillation	1.576	1.080 - 2.299	5.655	0.019
History of smoking	1.494	1.085 - 2.057	6.161	0.014
eGFR	0.976	0.966 - 0.987	20.265	0.000
NT-proBNP	1.000	1.000 – 1.000	106.191	0.000
GDF-15	1.000	1.000 – 1.000	129.383	0.000
Indexed LV mass	1.020	1.013 - 1.026	33.039	0.000
LV global longitudinal strain	1.194	1.156 - 1.233	120.047	0.000
Infarct LGE	3.196	2.337 - 4.371	53.776	0.000
Atypical (non-infarct LGE)	1.660	1.150 - 2.396	7.437	0.007

Abbreviations per previous tables.

Bradley J, *et al. Heart* 2024; 110:195–201. doi: 10.1136/heartjnl-2023-322857

Supplemental Table 9. Model features for nested models

	Original model					Model with GDF-15			
**Term**	**Hazard Ratio**	**95% confidence interval**	**Wald χ^2**	**p value**		**Hazard Ratio**	**95% confidence interval**	**Wald χ^2**	**p value**
Age	1.026	1.014 - 1.037	19.344	<0.0001		1.015	1.001 - 1.029	3.981	0.035
Diabetes	1.437	1.078 – 1.917	6.171	0.014		0.962	0.666 - 1.39	0.039	0.793
COPD	1.742	1.239 – 2.449	10.296	0.0015		1.579	1.055 - 2.365	4.503	0.025
ln (NT-proBNP)	1.275	1.118 – 1.455	13.455	0.0004		1.038	0.902 - 1.195	0.248	0.570
ln (GDF-15)	··	··	··	··		2.127	1.644 - 2.752	30.179	<0.0001
GLS	1.073	1.038 – 1.109	17.526	<0.0001		1.096	1.054 - 1.139	19.394	<0.001
Infarct LGE	1.560	1.196 – 2.036	10.851	0.0012		1.463	1.063 - 2.013	4.976	0.019
Myocardial ECV	1.083	1.044 – 1.122	18.844	<0.0001		1.081	1.036 - 1.128	12.019	<0.001

Abbreviations per previous tables.

Bradley J, *et al. Heart* 2024; 110:195–201. doi: 10.1136/heartjnl-2023-322857

Supplemental Table 9. Net reclassification indices for nested models

	**Estimate**	**95% confidence interval**
NRI	0.299	0.173 – 0.431
Pr (Up | Case)	0.213	0.150 – 0.280
Pr (Down | Case)	0.787	0.720 – 0.850
Pr (Down | Ctrl)	0.936	0.925 – 0.946
Pr (Up | Case)	0.064	0.054 – 0.075

Abbreviations per previous tables.

Bradley J, *et al. Heart* 2024; 110:195–201. doi: 10.1136/heartjnl-2023-322857

Supplemental Table 10. Univariable association of natural log-transformed GDF-15 and the composite primary outcome

**Heart Failure Stage**	**Hazard Ratio**	**95% confidence interval**	**Wald χ^2**	**p value**
A-B	4.242	2.880 – 6.250	53.436	<0.0001
C	3.039	2.424 – 3.811	92.763	<0.0001

Bradley J, *et al. Heart* 2024; 110:195–201. doi: 10.1136/heartjnl-2023-322857

Supplemental Table 11. Multivariable prognostic modelling for the composite outcome in HF sub-groups

	Heart Failure Stage A/B					Heart Failure Stage C			
**Term**	**Hazard Ratio**	**95% confidence interval**	**Wald χ^2**	**p value**		**Hazard Ratio**	**95% confidence interval**	**Wald χ^2**	**p value**
Age	1.024	0.997 - 1.052	3.186	0.082		1.011	0.994 - 1.029	1.590	0.21
COPD	3.888	1.700 - 8.891	11.043	0.002		1.240	0.736 - 2.088	0.670	0.415
ln (GDF-15)	2.970	1.774 - 4.973	18.292	<0.0001		2.210	1.678 - 2.911	32.587	<0.0001
GLS	1.101	1.025 - 1.184	7.382	0.010		1.093	1.046 - 1.142	15.902	<0.0001
Infarct LGE	1.796	0.872 - 3.698	2.690	0.109		1.389	0.938 - 2.056	2.756	0.100
Myocardial ECV	1.044	0.950 - 1.148	0.854	0.362		1.109	1.054 - 1.167	16.515	<0.0001

Abbreviations per previous tables.

Bradley J, *et al. Heart* 2024; 110:195–201. doi: 10.1136/heartjnl-2023-322857

Supplemental Table 12. Model performance

Heart Failure Stage	Discrimination	95% confidence interval
A-B	0.830	0.774 - 0.887
C	0.790	0.753 - 0.827

Bradley J, et al. Heart 2024; 110:195–201. doi: 10.1136/heartjnl-2023-322857

Supplemental Figure 1. Distribution GDF-15 (A) before and (B) after natural logarithmic transformation

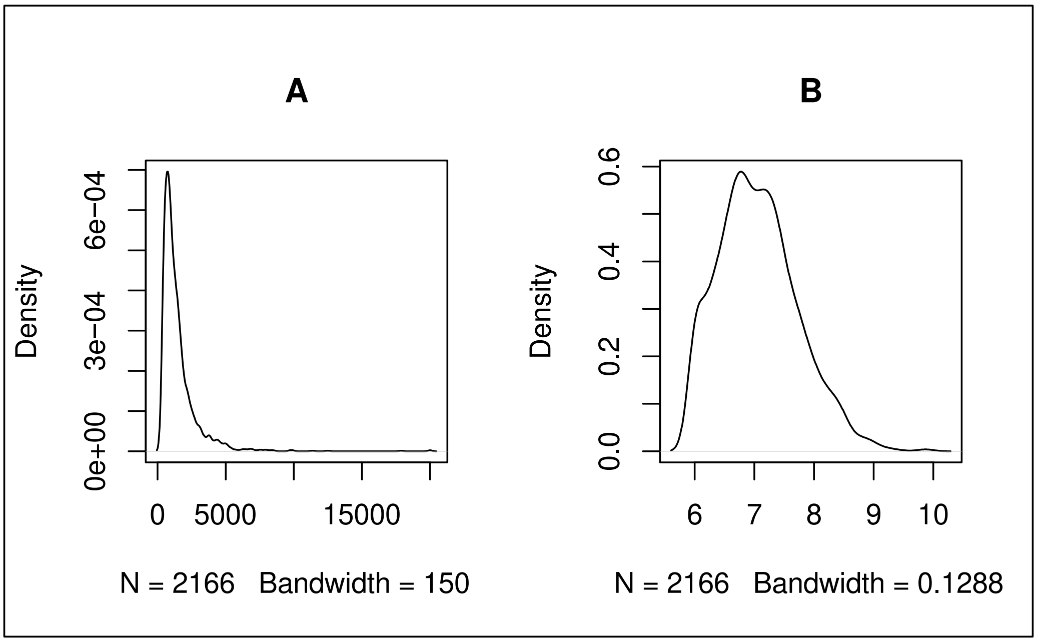

Bradley J, *et al. Heart* 2024; 110:195–201. doi: 10.1136/heartjnl-2023-322857

Supplemental Figure 2. Resampling calibration plot for Model 2 at 3 years (1095 days)

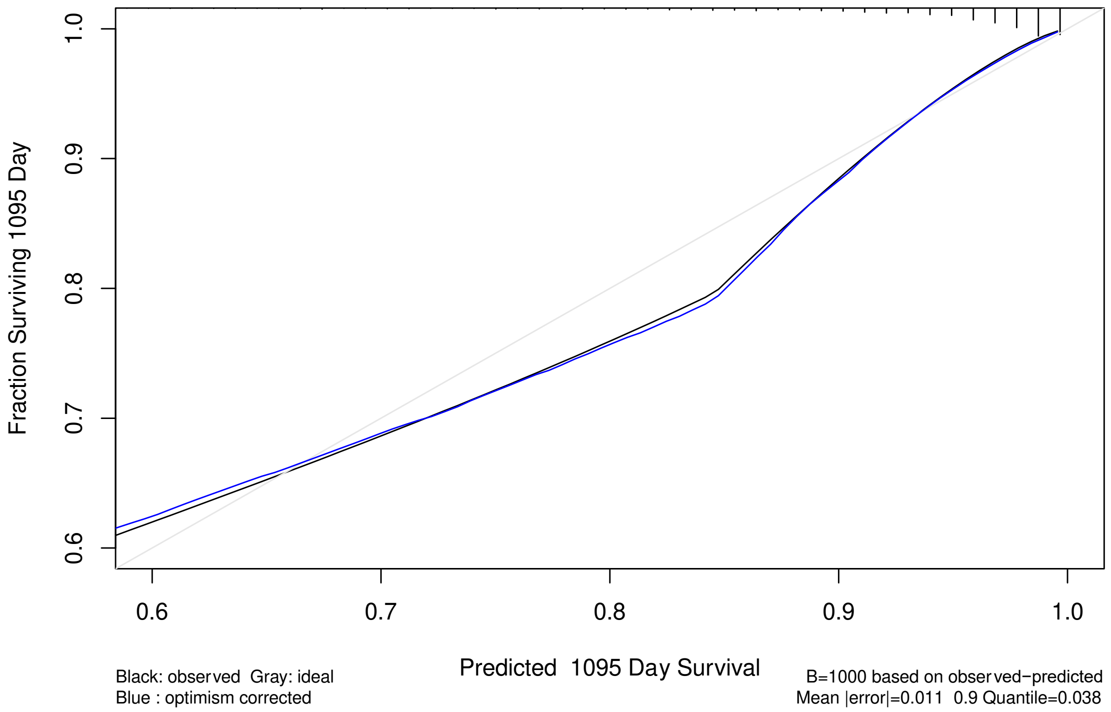

The smooth black line is the apparent calibration, and the blue line is the bootstrap optimism-(overfitting-) corrected calibration curve, both estimated by adaptive linear spline hazard regression. The grey line is the line of identity and represents perfect calibration. Mean |error| is equivalent to the Integrated Calibration Index and 0.9 quantile is equivalent to E_{90}.[19] A rug plot of the distribution of predicted outcome probabilities sits on the top axis of the plot. Survival is survival free of hospitalisation for heart failure or all-cause mortality.

As evidenced by the rug-plot across the top-axis, the majority of observations in the current investigation are relatively low-risk (predicted survival probability > 0.9). The Integrated Calibration Index (0.011) and E_{90} (0.038) both indicate a high degree of agreement between predicted and observed risk - mean difference between predicted and observed risk across all observations is 0.011, and for 90% observations this difference is less than 0.038.

The visualisation indicates a wide range of under-estimation at higher risk levels, although as demonstrated by the rug-plot, there were not many observations at these risk levels. Furthermore, a 7% or higher risk of heart failure hospitalisation or death in this low risk cohort represents high risk regardless of further breakdown. Underestimating risk in patients at still higher risk is likely to have little clinical impact.

Bradley J, *et al. Heart* 2024; 110:195–201. doi: 10.1136/heartjnl-2023-322857

Supplemental Figure 3. Decision Curve Analysis for nested modelling

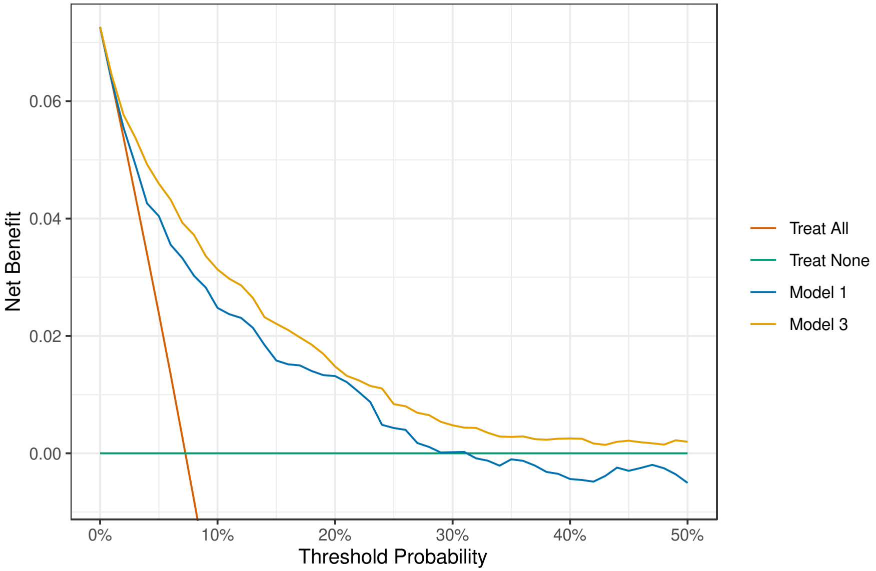

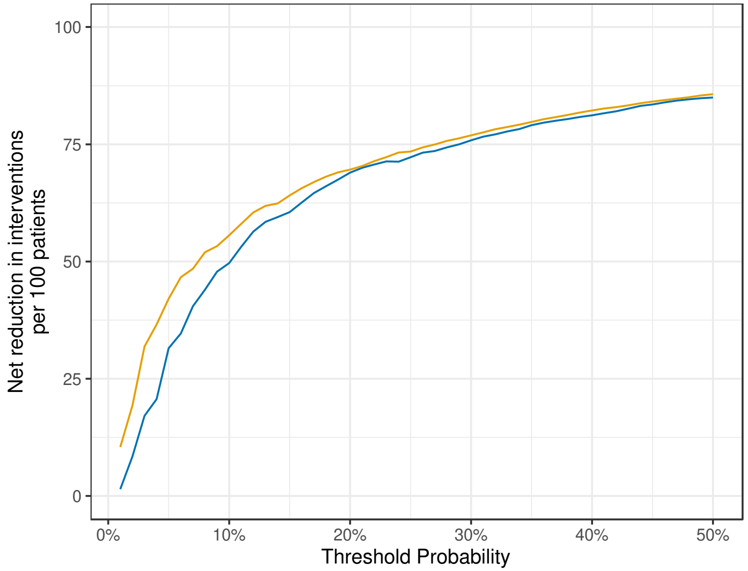

(Left) Decision curve analysis shows that risk prediction using the model that included GDF-15 (yellow line) leads to net clinical benefit at all decision thresholds (i.e., the threshold at which a decision regarding patient management, such as commencing an intervention to reduce risk, is taken) compared to the model that did not consider GDF-15 (blue). By convention, the default strategies of intervening for all patients (red) and intervening for none (green), are also shown. (Right) Furthermore, risk prediction using the model that included GDF-15 (yellow line) leads to a net reduction in the number of unnecessary interventions compared to the model that did not consider GDF-15 (blue).

Bradley J, *et al. Heart* 2024; 110:195–201. doi: 10.1136/heartjnl-2023-322857
